# Supplementary material for: Induced Mitochondrial Alteration and DNA Damage via IFNGR-JAK2-STAT1-PARP1 Pathway Facilitates Viral Hepatitis Associated Hepatocellular Carcinoma Aggressiveness and Stemness
Source: Cancers (Basel). 2021 Jun 2;13(11):2755. doi: 10.3390/cancers13112755 (PMC8199505; doi:10.3390/cancers13112755)
Supplement: Supplementary file 1 [file cancers-13-02755-s001.zip › cancers-1207722-supplementary.pdf]

SUPPLEMENTARY INFORMATION

Induced Mitochondrial Alteration and DNA damage via IFNGR-JAK2-STAT1-PARP1 Pathway Facilitates Viral Hepatitis Associated Hepatocellular Carcinoma Aggressiveness and Stemness

Yih-Giun Cherng[1,2], Yi Cheng Chu[3], Vijesh Kumar Yadav[4], Ting-Yi Huang[4], Ming-Shou Hsieh[4], Kwai-Fong Lee[5], Wei-Hwa Lee[6], Chi-Tai Yeh[4,7*] and Jiann Ruey Ong[8,9,10*]

1 Department of Anesthesiology, School of Medicine, College of Medicine, Taipei Medical University, Taipei, Taiwan.

2 Department of Anesthesiology, Taipei Medical University-Shuang Ho Hospital, New Taipei City, Taiwan.

3 Department of Medicine, St. George's University School of Medicine, St. George, Grenada

4 Department of Medical Research & Education, Taipei Medical University - Shuang Ho Hospital, New Taipei City, 235, Taiwan.

5 Biobank management center, Taipei Medical University-Shuang Ho Hospital, New Taipei City, Taiwan

6 Department of Pathology, Taipei Medical University-Shuang Ho Hospital, New Taipei City, Taiwan

7 Department of Medical Laboratory Science and Biotechnology, Yuanpei University of Medical Technology, Hsinchu 300, Taiwan.

8 Department of Emergency Medicine, Taipei Medical University-Shuang Ho Hospital, New Taipei City, Taiwan.

9 Graduate institute of injury prevention and control, Taipei Medical University, Taipei, Taiwan.

10 Department of Emergency Medicine, School of Medicine, Taipei Medical University, Taipei, Taiwan.

* Correspondence:

Dr. Chi-Tai Yeh, Ph.D. Department of Medical Research & Education, Taipei Medical University - Shuang Ho Hospital, New Taipei City, 235, Taiwan. No. 291, Zhongzheng Road, Zhonghe District, New Taipei City, Taiwan 235. Email: ctyeh@s.tmu.edu.tw Tel.: +886-2-2490088 ext. 8885

Dr. Jiann Ruey Ong, Ph. D. Department of Emergency Medicine, Shuang-Ho Hospital-Taipei Medical University, New Taipei City, Taiwan. Email: malsia95@gmail.com Tel.: +886-2-2490088 ext. 8885

Supplementary Table S1. Clinical details of HBV- and HCV-infected patients from the Taipei Medical University Shuang-Ho Hospital HCC cancer cohort.

Patient	Sample Type	Sex	Age	Serology
1	Normal	F	72	
2	Normal	F	73	
3	Normal	M	67	
4	Normal	F	52	
5	Normal	M	48	
6	Normal	M	63	
7	Normal	M	60	
8	Normal	M	49	
9	Normal	M	58	
10	Normal	M	62	
11	Primary Tumor	F	77	
12	Primary Tumor	F	78	
13	Primary Tumor	M	49	
14	Primary Tumor	M	56	
15	Primary Tumor	M	42	
16	Primary Tumor	M	38	
17	Primary Tumor	F	64	
18	Primary Tumor	M	81	
19	Primary Tumor	F	57	
20	Primary Tumor	M	68	
21	Primary Tumor	M	63	HCV
22	Primary Tumor	F	48	HBV
23	Primary Tumor	F	72	HCV
24	Primary Tumor	M	66	HBV
25	Primary Tumor	M	57	HBV
26	Primary Tumor	M	52	HBV
27	Primary Tumor	M	50	HBV+HCV
28	Primary Tumor	M	61	HBV+HCV
29	Primary Tumor	F	75	HCV
30	Primary Tumor	M	67	HCV

Supplementary Table S2. Commercial Antibodies list.

Targets	Analysis	Description	Kda	Dilution	Company
JAK2	IHC	Anti-JAK2 antibody (ab245303)		1:200	Abcam
JAK2	Western	Anti-JAK2 antibody (ab245303)	125	1:1000	Abcam
p-JAK2	Western	E-Cadherin (24E10) Rabbit mAb #3195	135	1:1000	Cell Signaling
STAT3	Western	Stat3 (D1B2J) Rabbit mAb #30835	86	1:1000	Cell Signaling
p-STAT3	Western	Phospho-Stat3 (Tyr705) Antibody #9131	86	1:1000	Cell Signaling
E-cadherin	Western	E-Cadherin (24E10) Rabbit mAb #3195	135	1:1000	Cell Signaling
N-cadherin	Western	N-Cadherin Antibody #4061	140	1:1000	Cell Signaling
Vimentin	Western	Vimentin (D21H3) XP® Rabbit mAb #5741	57	1:1000	Cell Signaling
β-actin	Western	Anti-beta Actin antibody (ab8227)	45	1:10000	Abcam
CD133	Western	Anti-CD133 antibody - Stem Cell Marker (ab19898)	133	1:1000	Abcam
KLF4	Western	Anti-KLF4 antibody (ab106629)	65	1:1000	Abcam
SOX2	Western	Anti-SOX2 antibody (ab97959)	35	1:1000	Abcam
Slug	Western	Anti-SLUG antibody (ab27568)	30	1:1000	Abcam
Bcl-xl	Western	Bcl-xL Antibody (H-5): sc-8392	30	1:1000	Santa Cruz
pro-/Cleaved-Caspase 7	Western	Caspase-7 Antibody #9494	35/30/20	1:1000	Cell Signaling
pro-/Cleaved-Caspase 9	Western	Caspase-9 Antibody (Human Specific) #9502	45/37/35	1:1000	Cell Signaling
pro-/Cleaved-PARP	Western	Cleaved PARP (Asp214) (D64E10) XP® Rabbit mAb #5625	116/89	1:1000	Cell Signaling

Supplementary Table S3. RT-PCR Primer sequences.

Gene	Forward Sequence (5' → 3')	Reverse Sequence (5' → 3')
JAK2	CAATGATAAACAAGGGCAAATGAT	CTTGGCAATCTTCCGTTGCT
CD133	GTCACCATTGACTTCTTGGTGCTGT	TGTCAGATGGAGTTACGCAGGTTTC
KLF4	CCTTTCAGTGCCAGAAGT	ACTACGTGGGATTTAAAAGTGC
SOX2	AGGGCTGGACTGCGAACTG	TTTGCACCCCTCCCAATTC
GAPDH	AATCCCATCACCATCTTCCAG	CACGATACCAAAGTTGTCATGG

Supplementary Table S4. Figure 1H IHC scoring matrix.

IHC Scoring Matrix

Normal			nvHCC			vHCC		
I	P	Q	I	P	Q	I	P	Q
1	5	5	2	30	60	3	100	300
2	15	30	2	15	30	3	90	270
2	50	100	1	60	60	3	70	210
2	30	60	2	40	80	2	90	180
1	25	25	2	70	140	2	75	150
1	80	80	2	50	100	2	60	120
2	30	60	3	25	75	2	70	140
1	10	10	2	20	40	1	90	90
1	80	80	1	45	45	1	80	80
1	75	75	1	75	75	2	60	120

***Intensity (I) x Positive Cells (P) = Quantitative Scoring (Q)**

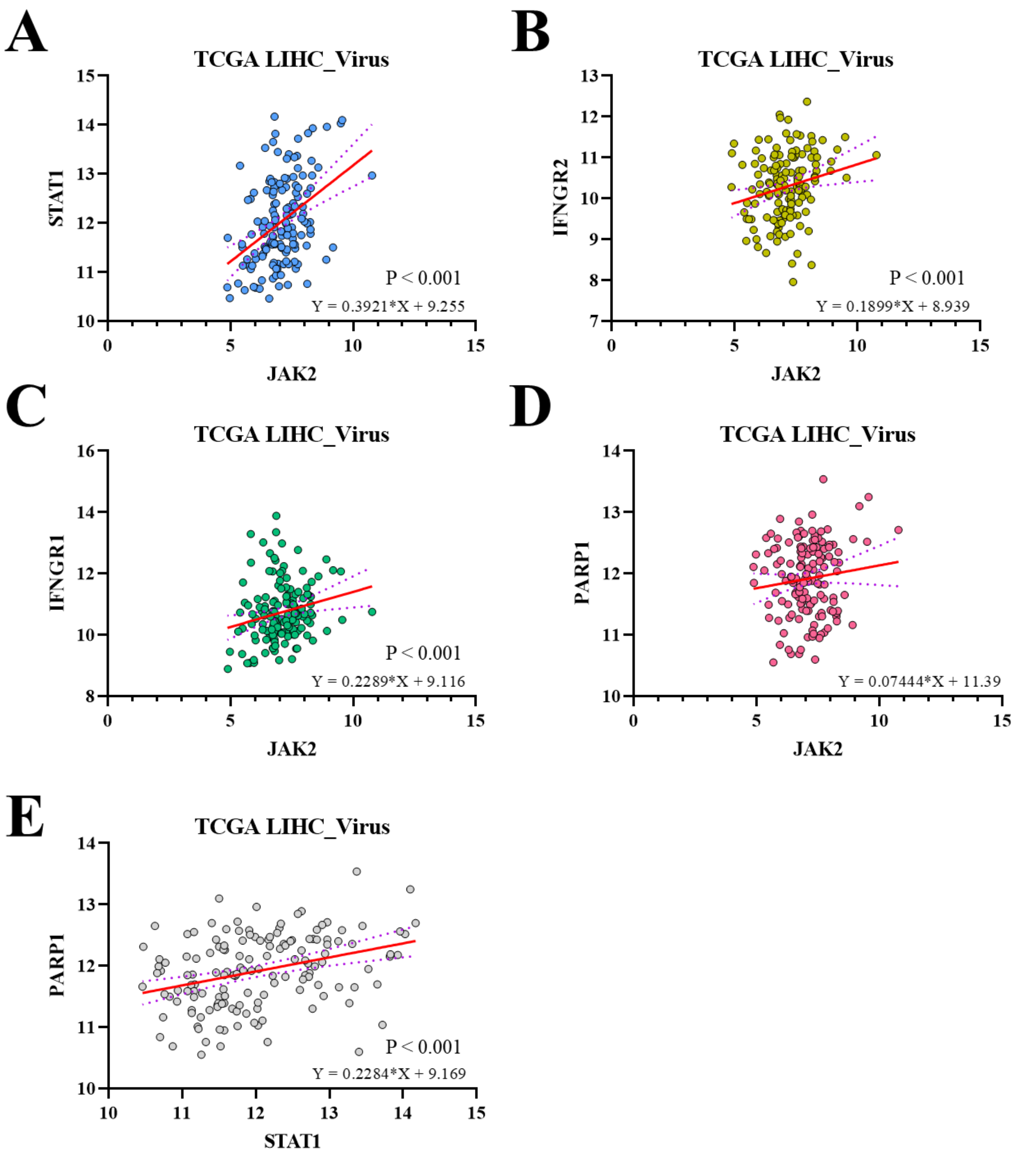

Supplementary Figure S1. Correlation of IFNGR-JAK2-STAT1-PARP1 axis in TCGA LIHC Virus

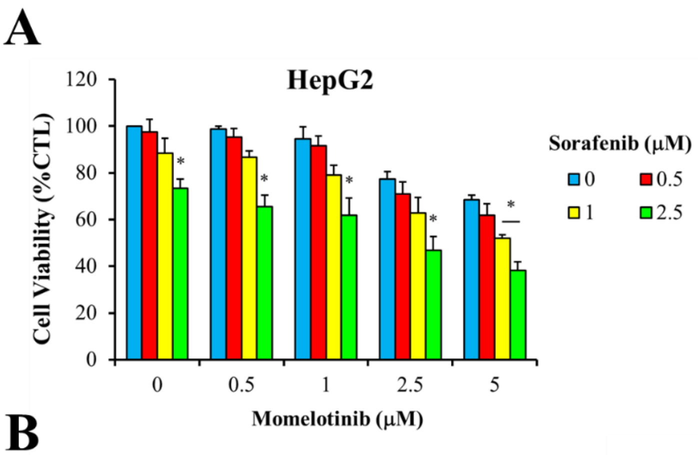

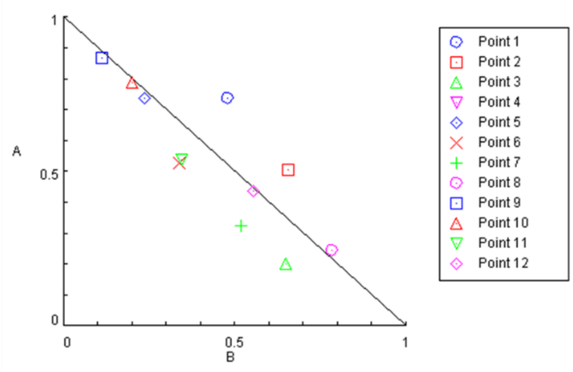

Symbol	Sorafenib	Momelotinib	Effect	CI
○	0.5	0.5	0.0476	1.21855
□	0.5	1.0	0.0845	1.16295
△	0.5	2.5	0.2918	0.85078
▽	0.5	5.0	0.3823	1.16581
◇	1.0	0.5	0.1339	0.97493
×	1.0	1.0	0.2107	0.86613
+	1.0	2.5	0.3708	0.84322
○	1.0	5.0	0.4809	1.03023
□	2.5	0.5	0.3445	0.97847
△	2.5	1.0	0.3806	0.99000
▽	2.5	2.5	0.5325	0.88401
◇	2.5	5.0	0.6171	0.99210

Supplementary Figure S2. Combination of momelotinib and sorafenib suppressed proliferation of nvHCC cells, HepG2.

SNU387

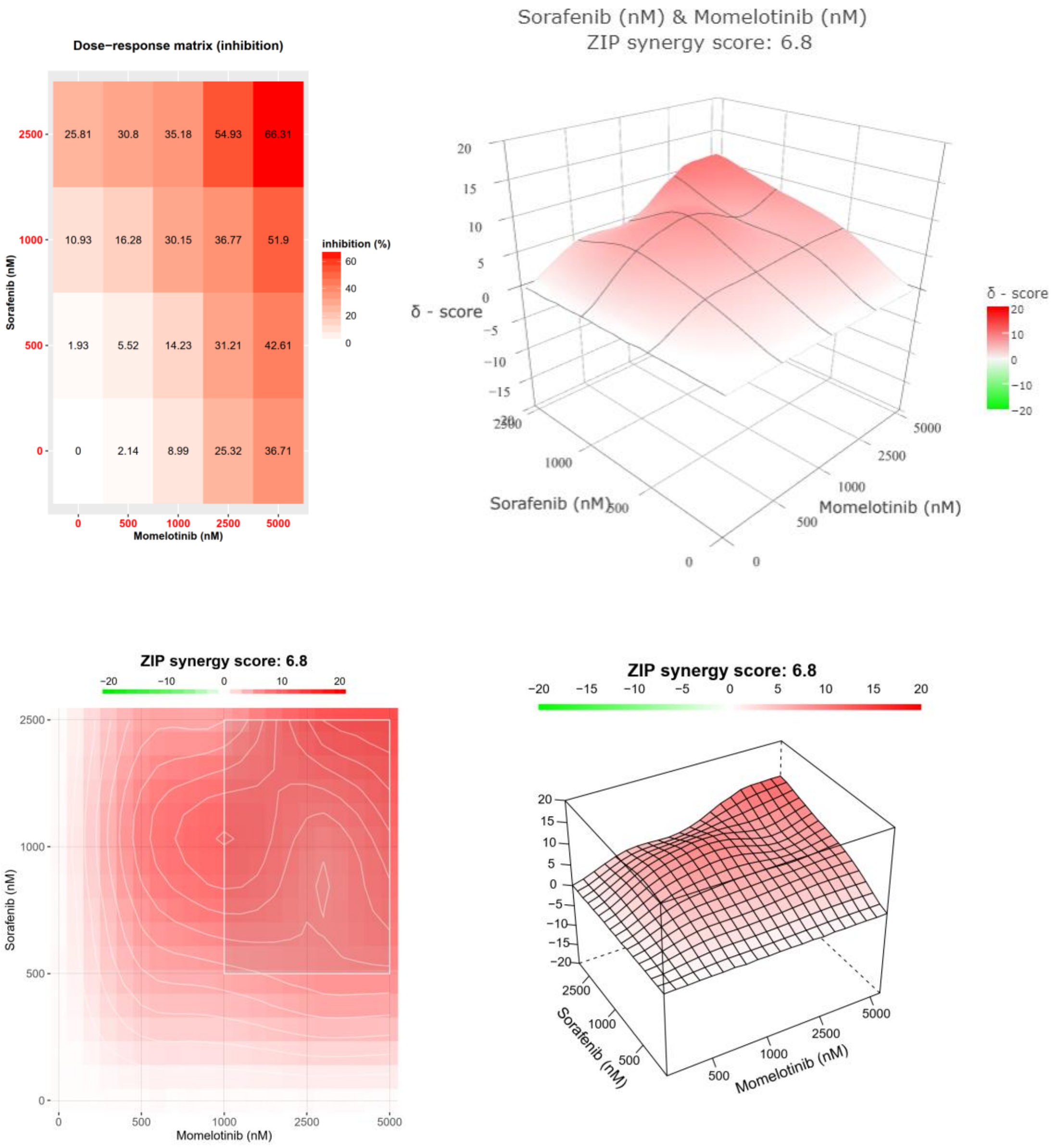

Supplementary Figure S3. Synergy score for combined Momelotinib and Sorafenib treatment with SNU387 cell.
(Viral liver cancer, image generated from SynergyFinder)

HepG2

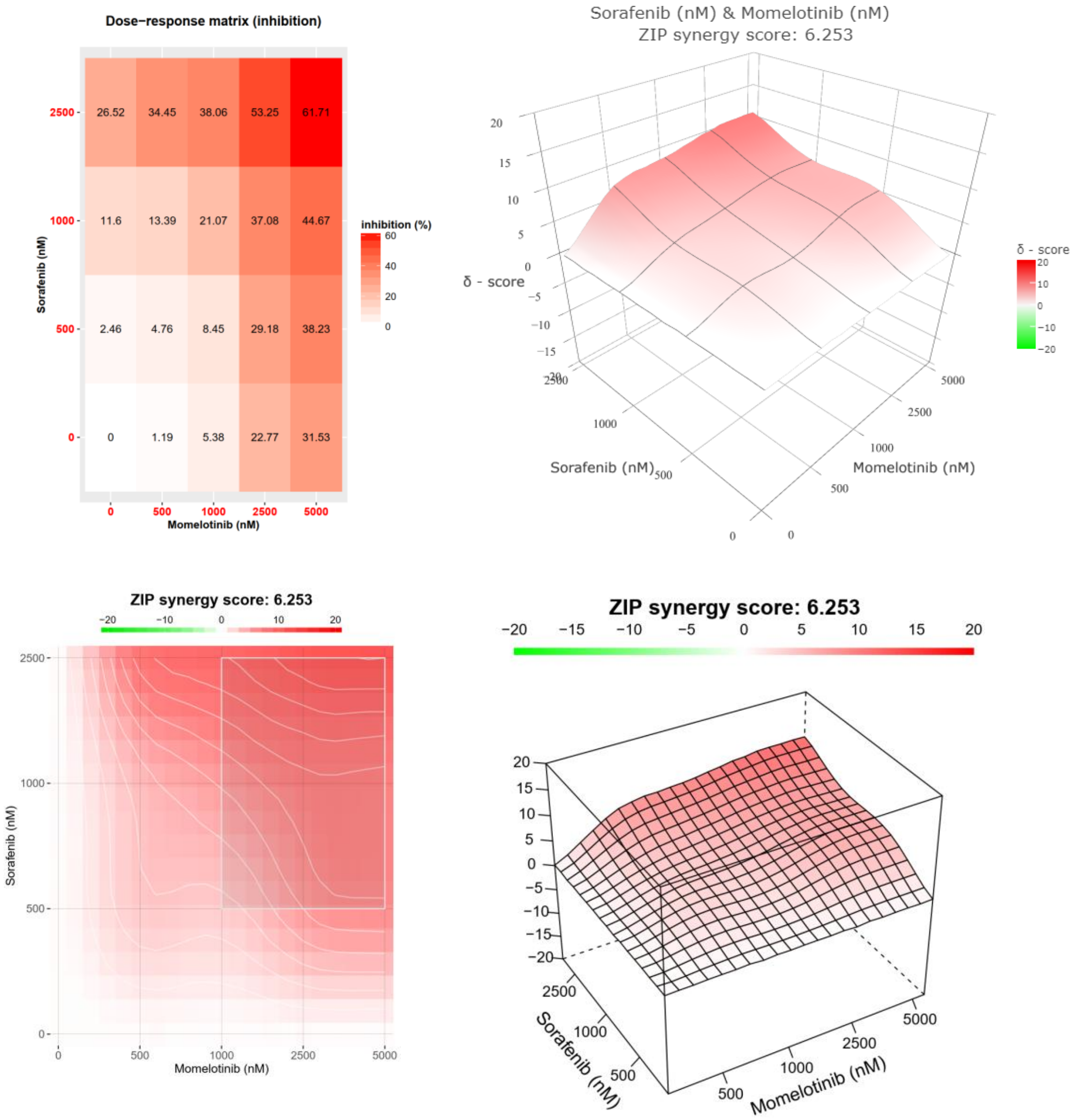

Supplementary Figure S4. Synergy score for combined Momelotinib and Sorafenib treatment with HepG2 cell. (Non-viral liver cancer, image generated from SynergyFinder)

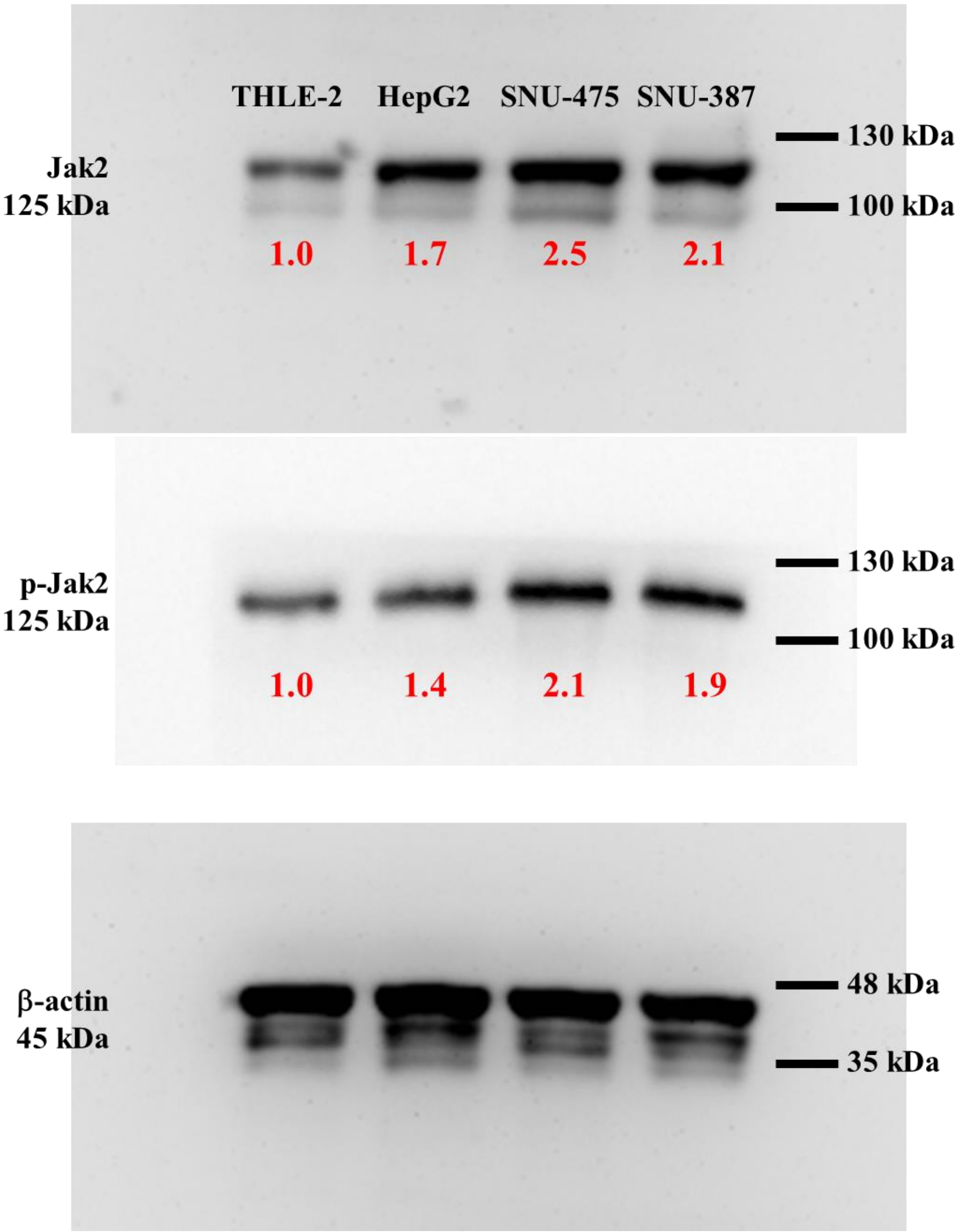

Supplementary Figure S5. Full-size blots of Figure 2B

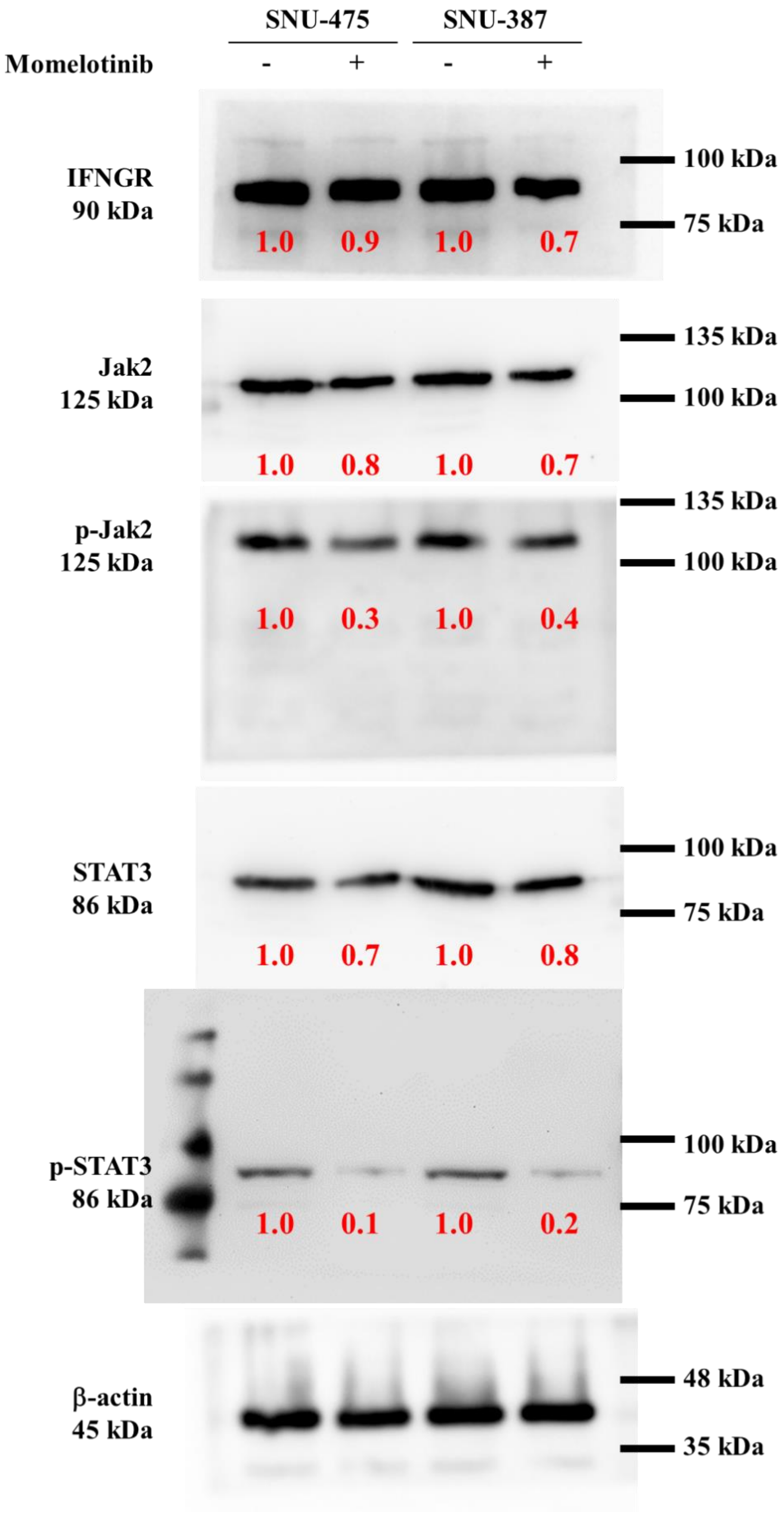

Supplementary Figure S6. Full-size blots of Figure 2E

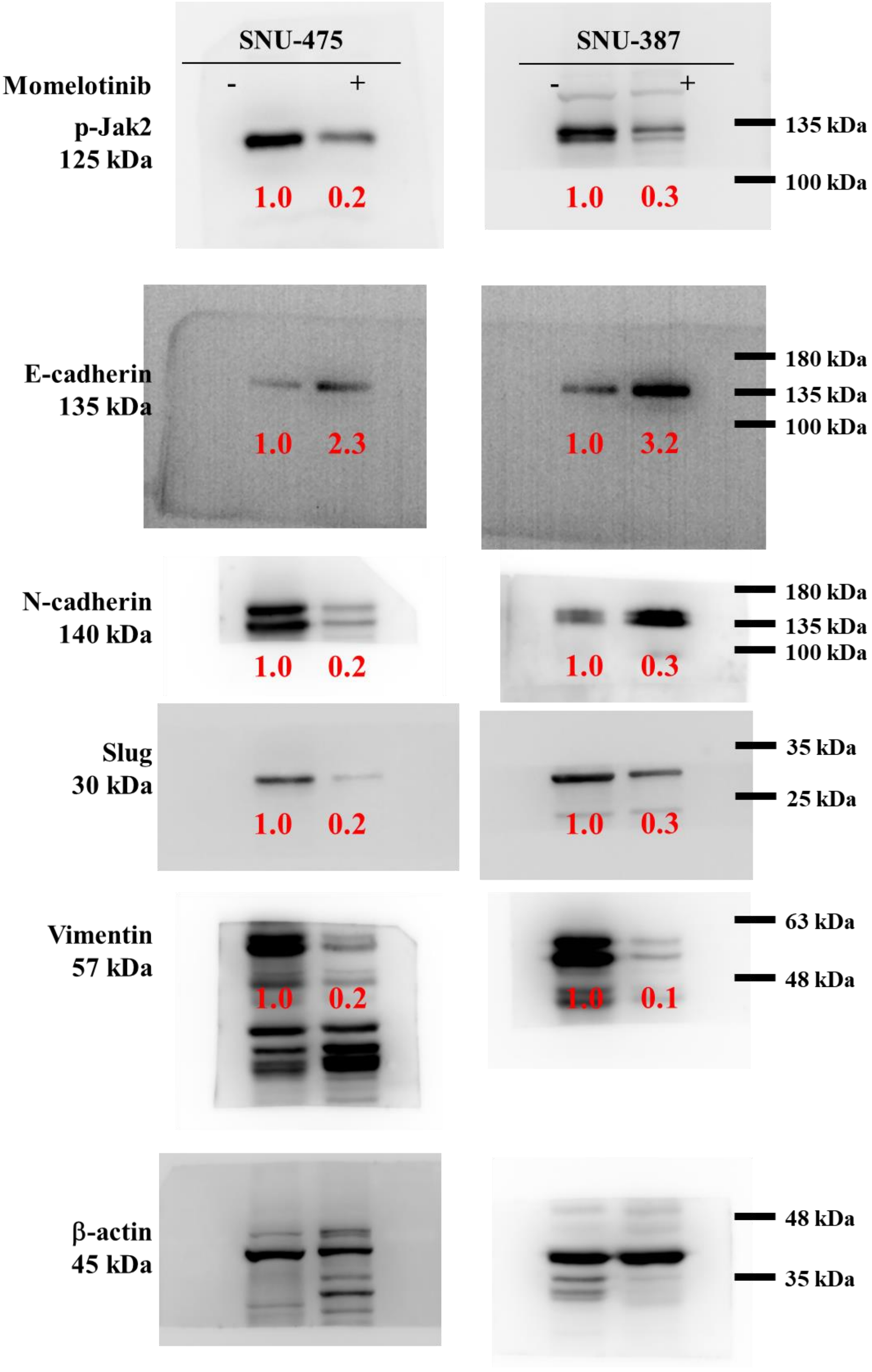

Supplementary Figure S7. Full-size blots of Figure 3D

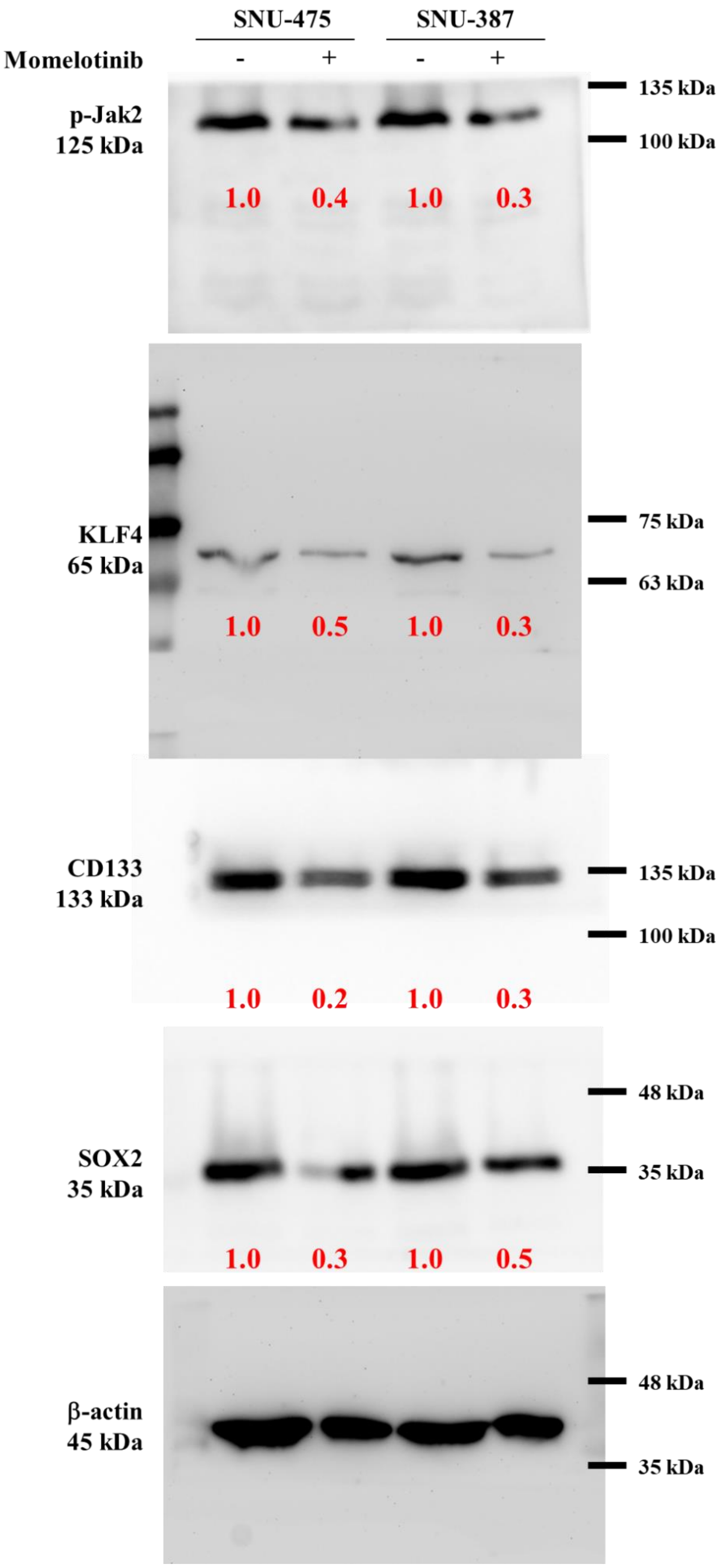

Supplementary Figure S8. Full-size blots of Figure 4C

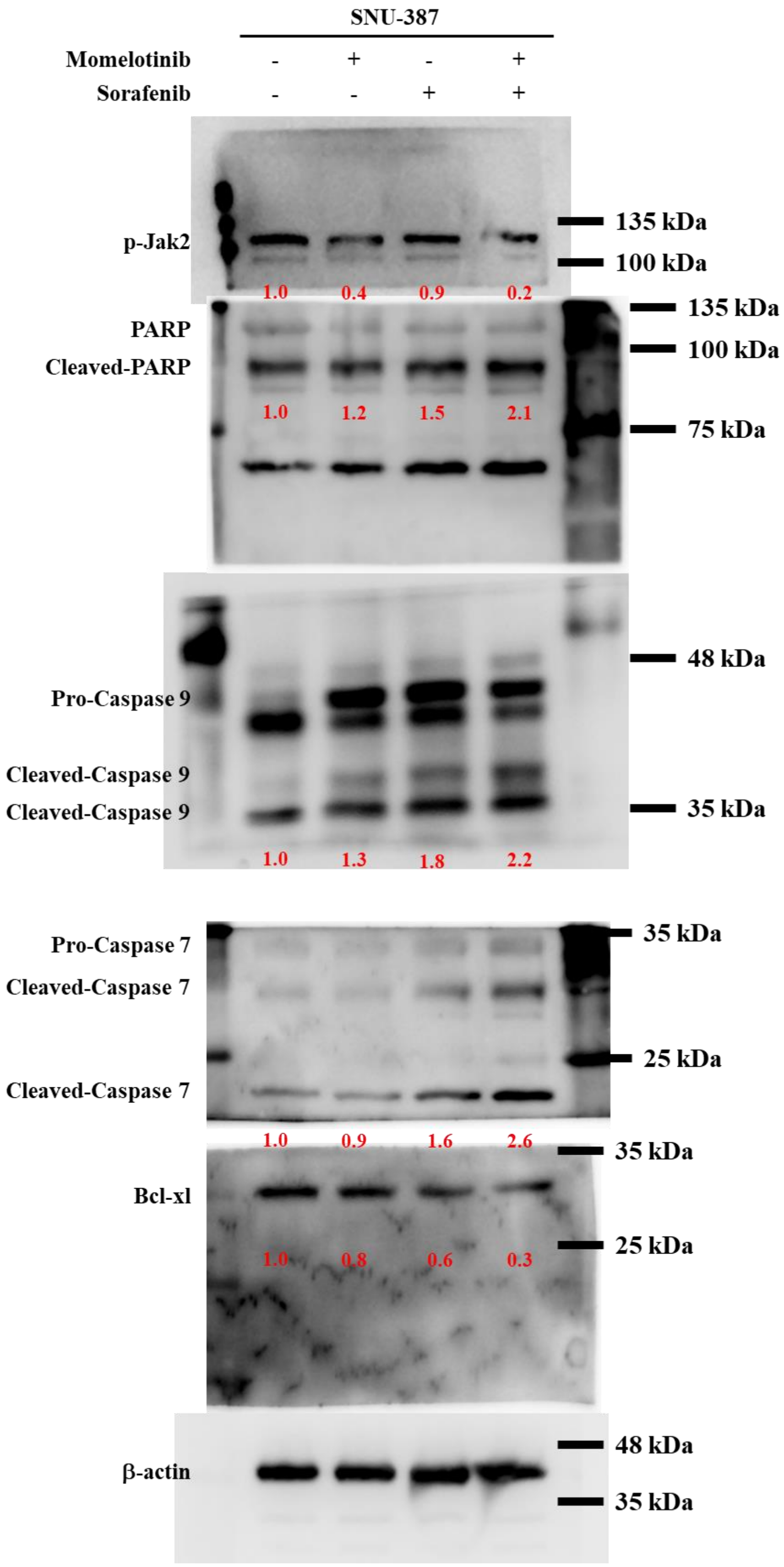

Supplementary Figure S9. Full-size blots of Figure 5D
